# Optimizing anesthesia strategies to NSCLC patients in VATS procedures: Insights from drug requirements and patient recovery patterns

**DOI:** 10.1515/med-2024-0961

**Published:** 2024-06-03

**Authors:** Linghui Kong, Hong Yin, Danran Zhou, Xin Li, Jie Zhou

**Affiliations:** Department of Pathology, Peking University Cancer Hospital (Inner Mongolia Campus) & Affiliated Cancer Hospital of Inner Mongolia Medical University, Hohhot, China; Department of Anesthesiology, Chengdu Fifth People’s Hospital, Chengdu, China; Department of Anesthesiology, Perioperative and Pain Medicine, Brigham and Women’s Hospital, Boston, MA, United States of America; Department of Anesthesiology, Hubei Cancer Hospital, Tongji Medical College Huazhong University of Science and Technology, Wuhan, China

**Keywords:** NSCLC, histological, chemotherapy, radiotherapy, epidural anesthesia, general anesthesia, rocuronium bromide, midazolam, post-anesthesia care unit, phenylephrine, remifentanil, hydromorphone, hospital stay

## Abstract

Understanding the intricate relationship between cancer clinicopathological features and anesthetics dosage is crucial for optimizing patient outcomes and safety during surgery. This retrospective study investigates this relationship in patients with non-small cell lung cancer (NSCLC) undergoing video-assisted thoracic surgery (VATS). A comprehensive analysis of medical records was undertaken for NSCLC patients who underwent VATS with intravenous compound inhalation general anesthesia. Patients were categorized based on histological, chemotherapy, radiotherapy, and epidural anesthesia factors. Statistical analysis was performed to compare the differences between the groups. The results revealed compelling insights. Specifically, patients with lung adenocarcinoma (LUAD) undergoing VATS exhibited higher dosages of rocuronium bromide and midazolam during general anesthesia, coupled with a shorter post-anesthesia care unit (PACU) stay compared to those with squamous cell carcinoma (sqCL). Furthermore, chemotherapy patients undergoing VATS demonstrated diminished requirements for phenylephrine and remifentanil in contrast to their non-chemotherapy counterparts. Similarly, radiotherapy patients undergoing VATS demonstrated a decreased necessity for rocuronium bromide compared to non-radiotherapy patients. Notably, patients who received epidural anesthesia in combination with general anesthesia manifested reduced hydromorphone requirements and prolonged hospital stays compared to those subjected to general anesthesia alone. In conclusion, the findings from this study indicate several important observations in diverse patient groups undergoing VATS. The higher dosages of rocuronium bromide and midazolam in LUAD patients point to potential differences in drug requirements among varying lung cancer types. Additionally, the observed shorter PACU stay in LUAD patients suggests a potentially expedited recovery process. The reduced anesthetic requirements of phenylephrine and remifentanilin chemotherapy patients indicate distinct responses to anesthesia and pain management. Radiotherapy patients requiring lower doses of rocuronium bromide imply a potential impact of prior radiotherapy on muscle relaxation. Finally, the combination of epidural anesthesia with general anesthesia resulted in reduced hydromorphone requirements and longer hospital stays, suggesting the potential benefits of this combined approach in terms of pain management and postoperative recovery. These findings highlight the importance of tailoring anesthesia strategies for specific patient populations to optimize outcomes in VATS procedures.

## 1 Introduction

General anesthesia is a surgical procedure that involves a variety of drugs to induce unconsciousness, amnesia, analgesia, and muscle relaxation [1]. Nondepolarizing neuromuscular blocking drugs (NMBDs) are commonly used in general anesthesia during surgical procedures to provide muscle relaxation, facilitate endotracheal intubation, and improve surgical conditions [2]. Rocuronium bromide is a widely used NMBD that has been shown to have several advantages over other NMBDs, including a fast onset of action, a relatively short duration of action, and a reversible mechanism of action [3]. Rocuronium bromide works by blocking the transmission of acetylcholine at the neuromuscular junction, leading to skeletal muscle paralysis and relaxation [3]. In addition to its neuromuscular blocking effects, rocuronium bromide has been shown to inhibit inflammation and pain by suppressing nitric oxide production and enhancing prostaglandin E2 synthesis in endothelial cells [4].

However, the pharmacokinetics of NMBDs can be altered in different disease conditions, including the presence of tumors. Tumors are specialized sites of inflammation with unique molecular and histological characteristics that distinguish them from their origin organs [5]. Inflammation has been linked to cancer and can affect the host body’s metabolic status [6]. Moreover, some studies have suggested that inflammation may contribute to the development and progression of cancer [7]. Despite this knowledge, there is currently no evidence regarding the potential relationship between cancer clinicopathological features and anesthetic dosage in intraoperative patients. This represents a critical knowledge gap, as the use of anesthetics and vasoactive drugs in cancer patients may need to be adjusted based on the tumor’s characteristics to ensure optimal outcomes and safety during surgery.

Non-small cell lung cancer (NSCLC), representing the predominant form of lung cancer, presents a diverse clinicopathological spectrum, incorporating various histological subtypes and genetic alterations [8]. The inherent heterogeneity within NSCLC, coupled with its prevalence, suggests a complex interplay that may influence responses to anesthesia [7]. Recent attention to the impact of anesthesia on cancer outcomes underscores the need for a more explicit rationale.

In alignment with this rationale, this study aims to provide a more profound understanding of the intricate relationship between cancer clinicopathological features and anesthetic dosage during intraoperative care. We hypothesize that the diverse clinicopathological features of NSCLC, including histological subtypes, genetic alterations, and prior treatments such as chemotherapy and radiotherapy, may contribute to variations in anesthetic requirements during video-assisted thoracic surgery (VATS) procedures. Consequently, this study aims to explore the potential relationship between cancer clinicopathological features and anesthetic dosage in intraoperative patients with NSCLC. The findings of this study could provide valuable insights into the use of anesthetics and vasoactive drugs in intraoperative patients with NSCLC, with the potential to enhance patient outcomes and safety during surgery.

## 2 Method

**Figure 1:**
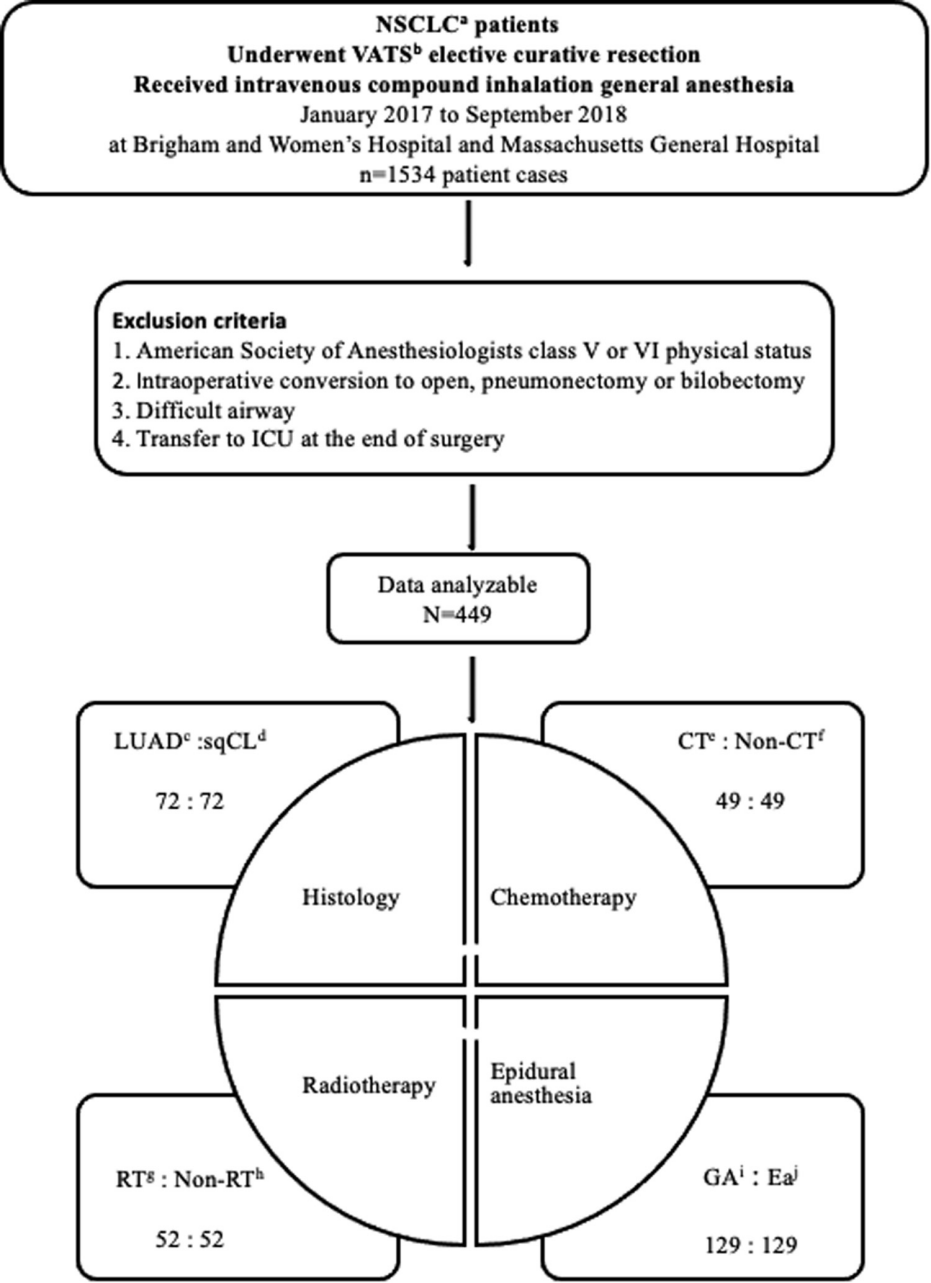
Study flow chart.

A retrospective study was performed to review the medical records of patients diagnosed with NSCLC who underwent VATS elective curative resection, including lobectomy, segmentectomy, and wedge resection. The procedures involved intravenous compound inhalation general anesthesia and were conducted at Brigham and Women’s Hospital and Massachusetts General Hospital from January 2017 to September 2018. The study protocol was approved by the institutional review board (IRB No 2019P00249). Subjects taking part in the research gave their informed consent. Patients’ data, including anesthesia records, surgery notes, and pathologic diagnoses, were examined to collect pertinent information for subsequent analysis, as illustrated in Figure 1.

### 2.1 Patient selection criteria

A total of 1,534 cases were included in the study based on the following criteria: (1) diagnosed with NSCLC, (2) underwent VATS elective curative resection (lobectomy, segmentectomy, or wedge resection), (3) received intravenous compound inhalation general anesthesia, and (4) availability of anesthesia records, surgery notes, and pathologic diagnosis. Patients were excluded if they met any of the following criteria: (1) American Society of Anesthesiologists class V or VI physical status, (2) intraoperative conversion to pneumonectomy or bilobectomy, (3) difficult airway, and (4) admission to the intensive care unit after surgery.

### 2.2 Cohort study information

Baseline information collected included demographic details, anesthesia techniques, surgery duration, and mechanical ventilation. Patients were categorized into distinct groups based on different studies. To ensure the comparability between the groups, a 1:1 matched cohort focused on clinicopathologic features was formed. The matching process incorporated various factors, including age, gender, anesthesia techniques, surgery duration, and mechanical ventilation. Propensity score analysis and logistic regression were employed to equate the groups based on demographic and clinical characteristics to minimize potential confounding factors. Subgroup analyses included: (1) Cancer histological type: lung adenocarcinoma (LUAD) group and squamous cell carcinomas (sqCL) group. (2) Chemotherapy: chemotherapy group (CT) and non-chemotherapy group (Non-CT). (3) Radiotherapy: radiation therapy (RT) group and non-radiation therapy (Non-RT) group. (4) Epidural anesthesia: epidural anesthesia combined with general anesthesia (EA) and general anesthesia (GA) alone.

### 2.3 Statistical analysis

Statistical analysis was performed using an ANOVA test to compare the two groups. Results were considered statistically significant if *p*-values were less than 0.05.

## 3 Results

1. LUAD patients undergoing VATS demonstrated higher dosages of rocuronium bromide and midazolam during general anesthesia and experienced a shorter post-anesthesia care unit (PACU) stay compared to sqCL patients.
Table 1 provides a summary of the study results, presenting the mean and standard deviation of anesthetic drugs, length of stay, and other relevant parameters for both the LUAD and sqCL groups. The analysis reveals no statistically significant differences in the requirement of phenylephrine, propofol, fentanyl, and neostigmine between the two groups (*P* > 0.05). However, the LUAD group demonstrated a significantly higher dosage of rocuronium bromide and midazolam during general anesthesia compared to the sqCL group (*P* < 0.05). Additionally, the LUAD patients undergoing VATS exhibited a shorter PACU stay than sqCL patients.
2. Chemotherapy patients undergoing VATS exhibited reduced requirements of phenylephrine and remifentanil compared to non-chemotherapy patients.
  Given the potential influence of prior or currently undergoing therapies, such as chemotherapy, to study the needs of intraoperative anesthetic and vasoactive drugs in patients with a history of or undergoing chemotherapy, another 1:1 matched cohort was performed by propensity score analysis and logistic regression. This cohort comprised 67 patients, each in the chemotherapy (CT) group and non-chemotherapy (Non-CT) group. The objective was to examine the requirements of intraoperative anesthetic drugs and vasoactive drugs. The analysis revealed no statistically significant differences between the two groups with respect to ephedrine, propofol, fentanyl, sufentanil, morphine, and rocuronium bromide (*P* > 0.05). However, patients in the CT group required lower amounts of phenylephrine and remifentanil compared to those in the non-CT group (*P* < 0.05). There were also no statistically significant differences in the length of hospital stay between the two groups (*P* > 0.05) (Table 2).
3. Radiotherapy patients undergoing VATS demonstrated a lower requirement for rocuronium bromide compared to non-radiotherapy patients.
  In order to study the needs for intraoperative anesthetic drugs and vasoactive drugs in patients with NSCLC undergoing radiotherapy, another 1:1 matched cohort was performed by propensity score analysis and logistic regression, including 52 patients each in the radiation therapy (RT) group and non-radiation therapy (Non-RT) group, respectively. There was no statistically significant difference between the two groups in terms of phenylephrine, propofol, fentanyl, remifentanil, sufentanil, hydromorphone, morphine, ephedrine, and length of hospital stay (*P* > 0.05). However, the RT group required less rocuronium bromide compared to the Non-RT group (*P* < 0.05). Furthermore, there were also no statistically significant differences in the duration of hospital stay between the two groups (*P* > 0.05) (Table 3).
4. Patients receiving epidural anesthesia in combination with general anesthesia exhibited reduced hydromorphone requirements and longer hospital stays compared to those receiving general anesthesia alone.

Epidural anesthesia is widely recognized as the standard for postoperative pain management in thoracic surgery, and it has been reported to have beneficial effects in surgical procedures. However, the occurrence of hypotension commonly occurs during epidural anesthesia combined with general anesthesia, leading anesthesiologists to administer more vasoactive drugs to maintain vital signs. The impact of epidural anesthesia on intraoperative vasoactive drugs, anesthetics, and other outcomes remains uncertain.

**Table 1:** Comparison of intraoperative drug dosage between LUAD and sqCL groups

	LUAD (*n* = 72)	sqCL (*n* = 72)	*F*	*P*
Phenylephrine	2711.26 ± 540.43	3125.56 ± 557.59	0.285	0.595
Propofol	507.54 ± 75.67	394.18 ± 55.12	1.466	0.228
Fentanyl	185.63 ± 7.97	162.92 ± 10.09	3.117	0.080
Hydromorphone	0.85 ± 0.08	0.66 ± 0.08	2.703	0.102
Midazolam	1.86 ± 0.14	1.38 ± 0.12	6.806	**0.010**
Neostigmine	7.44 ± 4.13	4.46 ± 1.64	0.449	0.504
Remifentanil	0.14 ± 0.07	0.19 ± 0.08	0.185	0.668
Rocuronium bromide	123.96 ± 8.26	88.51 ± 7.86	9.669	**0.002**
Sufentanil	1.99 ± 1.13	2.62 ± 1.29	0.134	0.715
Sugammadex	22.22 ± 9.58	37.00 ± 11.71	0.953	0.331
PACU (minutes)	432.02 ± 58.65	632.12 ± 66.93	5.078	**0.026**

Note. Data are expressed as mean ± standard deviation.

Abbreviations: LUAD, lung adenocarcinoma; sqCL, squamous cell carcinomas; PACU, post-anesthesia care unit.

Bold value indicates that these is significance between the groups.

**Table 2:** Comparison of intraoperative drug dosage between CT and Non-CT groups

	CT (*n* = 67)	Non-CT (*n* = 67)	*F*	*P*
Phenylephrine	1720.38 ± 301.96	4085.00 ± 767.73	8.307	**0.005**
Ephedrine	5.27 ± 1.65	9.94 ± 1.85	3.563	0.061
Propofol	358.22 ± 45.95	536.38 ± 83.59	3.488	0.064
Fentanyl	170.60 ± 10.48	187.39 ± 12.88	1.022	0.314
Remifentanil	0.06 ± 0.03	0.29 ± 0.10	5.259	**0.023**
Sufentanil	2.70 ± 1.23	2.07 ± 1.40	0.116	0.734
Morphine	0.12 ± 0.09	0.09 ± 0.09	0.053	0.818
Rocuronium bromide	94.15 ± 7.48	105.42 ± 8.62	0.977	0.325
Length of hospital stay (day)	5.83 ± 0.63	5.97 ± 0.41	0.034	0.853

Note. Data are expressed as mean ± standard deviation.

Abbreviations: CT, with chemotherapy; Non-CT, without chemotherapy.

Bold value indicates that these is significance between the groups.

**Table 3:** Comparison of intraoperative drug dosage between RT and Non-RT groups

	RT (*n* = 52)	Non-RT (*n* = 52)	*F*	*P*
Phenylephrine	2218.75 ± 374.70	3244.52 ± 725.88	1.577	0.212
Propofol	407.65 ± 61.14	546.73 ± 101.84	1.371	0.244
Fentanyl	162.02 ± 12.67	176.73 ± 11.75	0.725	0.396
Remifentanil	0.09 ± 0.04	0.26 ± 0.12	1.708	0.194
Sufentanil	3.26 ± 1.46	1.74 ± 1.57	0.499	0.481
Hydromorphone	0.70 ± 0.08	0.87 ± 0.11	1.599	0.209
Morphine	0.12 ± 0.12	0.00 ± 0.00	1.000	0.320
Rocuronium bromide	81.98 ± 9.00	120.43 ± 11.54	6.901	**0.010**
Ephedrine	5.54 ± 2.12	6.27 ± 1.68	0.073	0.787
Length of hospital stay (day)	6.43 ± 0.79	5.54 ± 0.37	1.059	0.306

Note. Data are expressed as mean ± standard deviation.

Abbreviations: RT, with radiotherapy; Non-RT, without radiotherapy.

Bold value indicates that these is significance between the groups.

To explore this potential relationship, the study divided patients into two groups: the EA group receiving current epidural anesthesia combined with general anesthesia and the GA group receiving general anesthesia alone. A1:1 matched cohort study was performed by propensity score analysis and logistic regression, including 129 patients each in the GA and EA group, respectively. Statistical analysis revealed no significant differences in the administration of phenylephrine, ephedrine, propofol, fentanyl, remifentanil, sufentanil, morphine, and rocuronium bromide between the two groups (*P* > 0.05). However, the hydromorphone requirement was lower in the GA group compared to the EA group (P < 0.05). Additionally, the GA group exhibited a longer duration of hospital stay compared to the EA group (*P* < 0.01) (Table 4).

**Table 4:** Comparison of intraoperative drug dosage between EA and GA groups

	EA (*n* = 129)	GA (*n* = 129)	*F*	*P*
Phenylephrine	2457.96 ± 353.30	2946.09 ± 398.63	0.841	0.360
Ephedrine	8.26 ± 1.23	6.21 ± 1.00	1.670	0.197
Propofol	353.02 ± 32.60	445.67 ± 48.29	2.529	0.113
Fentanyl	188.26 ± 7.22	167.32 ± 8.34	3.605	0.059
Remifentanil	0.14 ± 0.04	0.19 ± 0.05	0.455	0.501
Sufentanil	1.31 ± 0.58	3.22 ± 1.25	1.918	0.167
Hydromorphone	0.63 ± 0.59	0.85 ± 0.56	7.298	**0.007**
Morphine	0.03 ± 0.03	0.06 ± 0.05	0.287	0.593
Rocuronium bromide	98.16 ± 6.07	100.81 ± 5.48	0.105	0.746
Length of hospital stay (day)	6.12 ± 0.24	5.26 ± 0.30	4.821	**0.029**

Note. Data are expressed as mean ± standard deviation.

Abbreviations: EA, epidural anesthesia combined general anesthesia; GA, general anesthesia alone.

Bold value indicates that these is significance between the groups.

## 4 Discussion

Rocuronium bromide is a widely used neuromuscular blocking agent that was first introduced to the market in 1994 [9]. As a non-depolarizer, non-lipophilic neuromuscular blocking agent, rocuronium bromide acts by blocking the transmission of the nerve impulses at the neuromuscular junction [10]. It includes skeletal muscle relaxation, making it an invaluable tool in surgeries that require general anesthesia. By facilitating skeletal muscle relaxation, rocuronium bromide assists in various surgical procedures, such as facilitating endotracheal intubation, optimizing surgical access, and improving operating conditions for surgeons [3]. Rocuronium bromide has rapid onset action, intermediate duration of action, and adjustable depth of muscle relaxation [11]. The degree of muscle relaxation can be titrated by adjusting the dosage [12]. Midazolam is a medication belonging to the benzodiazepine class of drugs. It is commonly used as a sedative, anxiolytic (anti-anxiety), and amnestic agent in medical procedures [13,14]. Midazolam works by enhancing the effects of a naturally occurring neurotransmitter called gamma-aminobutyric acid in the brain, which helps to reduce anxiety, induce sedation, and promote relaxation [15]. Midazolam is frequently used as a component of general anesthesia to provide sedation and reduce anxiety before surgical procedures. It is often administered intravenously prior to the induction of anesthesia to help calm patients and facilitate the smooth transition into unconsciousness [16]. Midazolam can also cause temporary amnesia, which can be beneficial for patients who may experience distress or fear related to the surgery [17].

The PACU is a specialized area within a hospital or surgical center where patients are closely monitored and cared for immediately following a surgical procedure or the administration of anesthesia [18]. The length of stay in the PACU can vary depending on the type and complexity of the surgery, as well as the patient’s response to anesthesia and the surgical procedure [19].

The results of the present study show that LUAD patients undergoing VATS demonstrated higher dosages of rocuronium bromide and midazolam during general anesthesia and experienced a shorter PACU stay compared to sqCL patients. Radiotherapy patients undergoing VATS demonstrated a lower requirement for rocuronium bromide compared to non-radiotherapy patients. This could be attributed to the different proliferate metabolism of different tumor cells. This study provides another relevant finding that midazolam was increased in the LUAD patients as well.

Phenylephrine belongs to the class of sympathomimetic agents. It acts as a selective alpha-1 adrenergic agonist, which stimulates the alpha-1 receptors in the smooth muscles of blood vessels, leading to vasoconstriction [20]. By constricting blood vessels, phenylephrine increases blood pressure and can be used to treat hypotension during surgical procedures [21]. It is commonly used to maintain adequate blood pressure during anesthesia [21]. In our study, we found that chemotherapy patients undergoing VATS exhibited reduced requirements of phenylephrine and remifentanil compared to non-chemotherapy patients.

Hydromorphone is a potent opioid analgesic used for managing moderate to severe pain. It acts by binding to opioid receptors in the central nervous system, providing effective pain relief [22,23]. Proper use of hydromorphone requires close monitoring by healthcare professionals. They assess the patient’s pain levels, vital signs, and any adverse reactions or side effects. Dosage adjustments may be necessary based on the individual patient’s response and level of pain [24].

Most NSCLC operations are performed under general anesthesia alone. However, the additional epidural anesthesia has been used in multiple operation procedures, such as the operation of thoracic, abdominal, and delivery [25,26]. The main problem caused by epidural anesthesia is hypotension [27]. Some reports have shown that epidural anesthesia has potential benefits when combined with general anesthesia [28,29]. These benefits include perioperative pain relief, decreased consumption of anesthetic drugs and opioids, and reduced general anesthesia side effects [30,31,32]. Theoretically, it may include blocking afferent neural transmission from reaching the central nervous system, reducing the perioperative blood loss and vessel spasm, lowering the incidence of deep vein thrombosis, improving diaphragmatic function, and rapid postoperative recovery [33,34,35,36,37]. Our study showed that patients receiving epidural anesthesia in combination with general anesthesia exhibited reduced hydromorphone requirements and longer hospital stays compared to those receiving general anesthesia alone.

Our findings unveil potential explanations and clinical implications, shedding light on the intricate relationship between anesthetic requirements and specific clinicopathological features in NSCLC patients undergoing VATS. These insights carry significant relevance to current medical practice, potentially influencing patient outcomes and safety. NSCLC may have distinct clinicopathological features that make it more susceptible to the influence of anesthetics [38]. The hypoxic and inflammatory microenvironment of tumors, especially in NSCLC, could impact drug metabolism and responses [39,40,41]. The higher dosages of rocuronium bromide and midazolam in LUAD patients may be hypothesized to be related to specific characteristics of LUAD tumors, such as increased vascularity, altered drug clearance, tumor microenvironment, and metabolic variation [42,43,44,45]. Tumors can influence the local environment, potentially affecting the pharmacokinetics of anesthetic agents. It also might exhibit variations in neurotransmitter responses, influencing the dosage requirements for agents like midazolam. The interactions between anesthetic agents and tumor-related factors, such as the release of certain molecules from the tumor microenvironment, affect drug metabolism or response [46]. The higher dosages of rocuronium bromide and midazolam in LUAD patients may have clinical implications. This could suggest a need for personalized anesthetic approaches in lung cancer surgeries, tailoring drug dosages based on tumor histology to optimize patient responses.

Chemotherapy patients exhibiting reduced requirements of phenylephrine and remifentanil may be attributed to altered drug metabolism in the presence of chemotherapy agents [47,48]. Chemotherapy-induced changes in liver function or drug interactions might influence anesthetic needs [46]. Clinically, anesthesiologists should consider these variations when planning and administering anesthesia in patients with a history of or undergoing chemotherapy.

Lower doses of rocuronium bromide in radiotherapy patients could be linked to muscle changes induced by prior radiation. The impact of radiotherapy on muscle function and relaxation might affect the response to neuromuscular blocking agents [49]. Anesthesiologists should be aware of these considerations in the preoperative assessment of patients with a history of radiotherapy. Both chemotherapy and radiotherapy can elicit inflammatory responses and could play a role in altering the pharmacodynamics of rocuronium bromide [50]. They could also induce changes in muscle sensitivity, which leads to a reduced requirement for muscle relaxants during VATS procedures.

Reduced hydromorphone requirements and longer hospital stays in the epidural anesthesia group might be explained by the synergistic pain relief effects of epidural anesthesia, potentially leading to decreased opioid needs postoperatively [51]. The observed differences underscore the importance of moving toward personalized anesthesia approaches, considering individual patient characteristics, tumor histology, and prior treatments. This aligns with the current trend in precision medicine, emphasizing tailored medical interventions based on patient-specific factors. Understanding the impact of epidural anesthesia on hydromorphone requirements emphasizes the potential role of this combined approach in optimizing pain management strategies. This has implications for postoperative care and patient comfort. LUAD patients experiencing a shorter PACU stay suggest a potentially faster recovery process. This finding may contribute to enhanced patient outcomes, reducing the time spent in the immediate postoperative period. The variations in drug requirements among chemotherapy and radiotherapy patients necessitate considerations in anesthetic planning to ensure patient safety and effective pain management. Awareness of these factors may impact intraoperative and postoperative care decisions.

Despite providing valuable insights, our study has limitations. A single-center retrospective design limits generalizability. Further research should involve multicenter collaborations, diverse patient populations, and various cancer types to enhance external validity. Prospective studies, randomized controlled trials, and longitudinal studies are warranted for a more robust understanding and to explore causality. Potential avenues include large-scale multicenter studies, mechanistic investigations into anesthetic variations, and exploration of molecular and genetic biomarkers. Considering evolving cancer therapies, research should explore the impact of novel treatments on anesthetic needs. Emphasizing patient-centered outcomes, exploring Enhanced Recovery After Surgery protocols, and assessing the holistic impact of comprehensive interventions on NSCLC patients represent promising directions for future research.

## 5 Conclusions

In summary, our findings from the study indicate several important observations in different patient groups undergoing VATS, revealing notable variations in anesthetic requirements and outcomes. The higher dosages of rocuronium bromide and midazolam in LUAD patients point to potential differences in drug requirements among varying lung cancer types. Additionally, the observed shorter PACU stay in LUAD patients suggests a potentially expedited recovery process, emphasizing the clinical significance of tailoring anesthetic strategies. Furthermore, the reduced anesthetic requirements of phenylephrine and remifentanil in chemotherapy patients indicate distinct responses to anesthesia and pain management. Radiotherapy patients requiring lower doses of rocuronium bromide imply a potential impact of prior radiotherapy on muscle relaxation, adding a layer of consideration in anesthetic planning. Finally, the combination of epidural anesthesia with general anesthesia resulted in reduced hydromorphone requirements and longer hospital stays, suggesting the potential benefits of this combined approach in terms of pain management and postoperative recovery. These findings highlight the importance of tailoring anesthesia strategies for specific patient populations to optimize outcomes in VATS procedures.

Collectively, our observations stress the imperative need to tailor anesthesia strategies to the specific characteristics of patient populations, acknowledging the nuanced differences in drug requirements and recovery trajectories. Such tailored approaches have the potential to optimize outcomes in VATS procedures, paving the way for more individualized and effective perioperative care.

## References

[1] Brown EN, Lydic R, Schiff ND. General anesthesia, sleep, and coma. N Engl J Med. 2010;363:2638–50. 10.1056/NEJMra0808281 PMC3162622 21190458

[2] Clar DT, Liu M. Nondepolarizing neuromuscular blockers. In: StatPearls. Treasure Island (FL): StatPearls Publishing; 2024. 30521249

[3] Jain A, Wermuth HR, Dua A, Singh K, Maani CV. Rocuronium. In: StatPearls. Treasure Island (FL): StatPearls Publishing; 2023. 30969710

[4] Baek SB, Shin MS, Han JH, Moon SW, Chang B, Jeon JW, et al. Rocuronium bromide inhibits inflammation and pain by suppressing nitric oxide production and enhancing prostaglandin E2 synthesis in endothelial cells. Int Neurourol J. 2016;20:296–303. 10.5213/inj.1632796.398 PMC5209582 28043117

[5] Craig RG, Hunter JM. Neuromuscular blocking drugs and their antagonists in patients with organ disease. Anaesthesia. 2009;64(Suppl 1):55–65. 10.1111/j.1365-2044.2008.05871.x 19222432

[6] Chen L, Deng H, Cui H, Fang J, Zuo Z, Deng J, et al. Inflammatory responses and inflammation-associated diseases in organs. Oncotarget. 2017;9:7204–18. 10.18632/oncotarget.23208 PMC5805548 29467962

[7] Singh N, Baby D, Rajguru JP, Patil PB, Thakkannavar SS, Pujari VB. Inflammation and Cancer. Ann Afr Med. 2019;18:121–6. 10.4103/aam.aam_56_18 PMC6704802 31417011

[8] Chen Z, Fillmore CM, Hammerman PS, Kim CF, Wong KK. Non-small-cell lung cancers: a heterogeneous set of diseases. Nat Rev Cancer. 2014;14:535–46. 10.1038/nrc3775 PMC5712844 25056707

[9] Naguib M. Neuromuscular effects of rocuronium bromide and mivacurium chloride administered alone and in combination. Anesthesiology. 1994;81:388–95. 10.1097/00000542-199408000-00017 8053589

[10] Khuenl-Brady KS, Sparr H. Clinical pharmacokinetics of rocuronium bromide. Clin-Pharmacokinet. 1996;31:174–83. 10.2165/00003088-199631030-00002 8877248

[11] Lee S, Ro YJ, Koh WU, Nishiyama T, Yang HS. The neuromuscular effects of rocuronium under sevoflurane-remifentanil or propofol-remifentanil anesthesia: A randomized clinical comparative study in an Asian population. BMC Anesthesiol. 2016;16:65. 10.1186/s12871-016-0231-0 PMC4994310 27549387

[12] Kumar A, Suchetha S. Comparision of intubating conditions at 60 seconds with different doses of rocuronium using the timing principle. J Anesth Clin Res. 2018;9:4.

[13] Wright SW, Chudnofsky CR, Dronen SC, Wright MB, Borron SW. Midazolam use in the emergency department. Am J Emerg Med. 1990;8:97–100. 10.1016/0735-6757(90)90192-3 2302291

[14] Dundee JW, Halliday NJ, Harper KW, Brogden RN. Midazolam. A review of its pharmacological properties and therapeutic use. Drugs. 1984;28:519–43. 10.2165/00003495-198428060-00002 6394264

[15] Olkkola KT, Ahonen J. Midazolam and other benzodiazepines. Handb Exp Pharmacol. 2008;182:335–60. 10.1007/978-3-540-74806-9_16 18175099

[16] Mihalj M, Karlović Z, Vladić-Spaić D, Matić B, Mikulić I, Mikulić V, et al. Effects of midazolam co-induction to general anesthesia: A randomized clinical trial. Medicine (Baltimore). 2022;101:e31400. 10.1097/MD.0000000000031400 PMC9666170 36397390

[17] Alizadeh A, Naseri M, Ravanshad Y, Sorouri S, Banihassan M, Azarfar A. Use of sedative drugs at reducing the side effects of voiding cystourethrography in children. J Res Med Sci. 2017;22:42. 10.4103/1735-1995.202139 PMC5393102 28465701

[18] Wang X-L, He M, Feng Y. Handover patterns in the PACU: A review of the literature. J Perianesth Nurs. 2021;36:136–41. 10.1016/j.jopan.2020.05.005 33168405

[19] Abebe B, Kifle N, Gunta M, Tantu T, Wondwosen M, Zewdu D. Incidence and factors associated with post-anesthesia care unit complications in resource-limited settings: An observational study. Health Sci Rep. 2022;5:e649. 10.1002/hsr2.649 PMC9125872 35620534

[20] Al-Khrasani M, Karadi DA, Galambos AR, Sperlagh B, Vizi ES. The pharmacological effects of phenylephrine are indirect, mediated by noradrenaline release from the cytoplasm. Neurochem Res. 2022;47:3272–84. 10.1007/s11064-022-03681-2 PMC9546997 35945308

[21] Ko MJ, Kim H, Lee H, Park YH, Bang JY, et al. Effect of phenylephrine infusion on hypotension induced by the beach chair position. Medicine (Baltimore). 2020;99:e20946. 10.1097/MD.0000000000020946 PMC7360254 32664094

[22] Bao YJ, Hou W, Kong XY, Lee SJ, Park YH, Bang JY, et al. Hydromorphone for cancer pain. Cochrane Database Syst Rev. 2016;10:CD011108. 10.1002/14651858.CD011108.pub2 PMC6457981 27727452

[23] Suzan E, Eisenberg E, Treister R, Haddad M, Pud D. A negative correlation between hyperalgesia and analgesia in patients with chronic radicular pain: is hydromorphone therapy a double-edged sword? Pain Phys. 2013;16:65–76. 23340535

[24] Abs R, Verhelst J, Maeyaert J, Van Buyten JP, Opsomer F, Adriaensen H, et al. Endocrine consequences of long-term intrathecal administration of opioids. J Clin Endocrinol Metab. 2000;85:2215–22. 10.1210/jcem.85.6.6615 10852454

[25] Schlesinger T, Weibel S, Steinfeldt T, Sitter M, Meybohm P, Kranke P. Intraoperative management of combined general anesthesia and thoracic epidural analgesia: A survey among German anesthetists. Acta Anaesthesiol Scand. 2021;65:1490–6. 10.1111/aas.13971 34383293

[26] Bardia A, Sood A, Mahmood F, Orhurhu V, Mueller A, Montealegre-Gallegos M, et al. Combined epidural-general anesthesia vs general anesthesia alone for elective abdominal aortic aneurysm repair. JAMA Surg. 2016;151:1116–23. 10.1001/jamasurg.2016.2733 27603002

[27] Holte K, Foss NB, Svensén C, Lund C, Madsen JL, Kehlet H. Epidural anesthesia, hypotension, and changes in intravascular volume. Anesthesiology. 2004;100:281–6. 10.1097/00000542-200402000-00016 14739801

[28] Zanjani AP, Ghorbani A, Eslami B, Mirzashahi B. Epidural anesthesia combined with light general anesthesia for a juvenile with Charcot-Marie-Tooth disease undergoing corrective spine surgery: A case report. Anesth Pain Med. 2017;7:e14189. 10.5812/aapm.14189 PMC5903216 29696112

[29] Cakmakkaya OS, Kolodzie K, Apfel CC, Pace NL. Anaesthetic techniques for risk of malignant tumour recurrence. Cochrane Database Syst Rev. 2014;11:CD008877. 10.1002/14651858.CD008877.pub2 PMC10523187 25379840

[30] Blake DW. The general versus regional anaesthesia debate: time to re-examine the goals. Aust N Z J Surg. 1995;65:51–6. 10.1111/j.1445-2197.1995.tb01748.x 7818424

[31] Thepsoparn M, Sereeyotin J, Pannangpetch P. Effects of combined lower thoracic epidural/general anesthesia on pain control in patients undergoing elective lumbar spine surgery: A randomized controlled trial. Spine (Phila Pa 1976). 2018;43:1381–5. 10.1097/BRS.0000000000002662 29624542

[32] Du Y-T, Li Y-W, Zhao B-J, Guo XY, Feng Y, Zuo MZ, et al. Long-term survival after combined epidural-general anesthesia or general anesthesia alone: Follow-up of a randomized trial. Anesthesiology. 2021;135:233–45. 10.1097/ALN.0000000000003835 34195784

[33] Yang B, Qian F, Li W, Li Y, Han Y. Effects of general anesthesia with or without epidural block on tumor metastasis and mechanisms. Oncol Lett. 2018;15:4662–8. 10.3892/ol.2018.7870 PMC5835878 29541238

[34] Lee Y-C, Park S-J, Kim J-S, Cho CH. Effect of tranexamic acid on reducing postoperative blood loss in combined hypotensive epidural anesthesia and general anesthesia for total hip replacement. J Clin Anesth. 2013;25:393–8. 10.1016/j.jclinane.2013.02.006 23965206

[35] Kang J, Lin W, Wang H, Liang Y, Yu Z. Effects of general anesthesia and epidural anesthesia on deep vein thrombosis and perioperative cognitive function of patients undergoing total knee arthroplasty. Am J Transl Res. 2022;14:4786–94. PMC9360839 35958440

[36] Oh YJ, Lee JR, Choi YS, Koh SO, Na S. Randomized controlled comparison of combined general and epidural anesthesia versus general anesthesia on diaphragmatic function after laparoscopic prostatectomy. Minerva Anestesiol. 2013;79:1371–80. 23857436

[37] Schwarz SKW, Wong CL, McDonald WN. Spontaneous recovery from a spinal epidural hematoma with atypical presentation in a nonagenarian. Can J Anaesth. 2004;51:557–61. 10.1007/BF03018397 15197117

[38] Zhu W, Li S, Ji X, Tan H. Impact of anesthetic factors on prognosis of patients with non-small cell lung cancer after surgery. J Thorac Dis. 2023;15:4869–84. 10.21037/jtd-22-1812 PMC10586941 37868870

[39] Tan Z, Xue H, Sun Y, Zhang C, Song Y, Qi Y. The role of tumor inflammatory microenvironment in lung cancer. Front Pharmacol. 2021;12:688625. 10.3389/fphar.2021.688625 PMC8166205 34079469

[40] Roy S, Kumaravel S, Sharma A, Duran CL, Bayless KJ, Chakraborty S. Hypoxic tumor microenvironment: Implications for cancer therapy. Exp Biol Med (Maywood). 2020;245:1073–86. 10.1177/1535370220934038 PMC7400722 32594767

[41] Zhang D, Leal AS, Rous FA, Liby KT. Profiling changes in metabolism and the immune microenvironment in lung tumorigenesis. Ann Transl Med. 2019;7:S90. 10.21037/atm.2019.04.33 PMC6685892 31576298

[42] Li J, Gu X, Wan G, Wang Y, Chen K, Chen Q, et al. Rocuronium bromide suppresses esophageal cancer via blocking the secretion of C–X–C motif chemokine ligand 12 from cancer associated fibroblasts. J Transl Med. 2023;21:248. 10.1186/s12967-023-04081-y PMC10082495 37029408

[43] Magorian T, Wood P, Caldwell J, Fisher D, Segredo V, Szenohradszky J, et al. The pharmacokinetics and neuromuscular effects of rocuronium bromide in patients with liver disease. Anesth Analg. 1995;80:754–9. 10.1097/00000539-199504000-00018 7893030

[44] Neupane B, Pandya H, Pandya T, Austin R, Spooner N, Rudge J, et al. Inflammation and cardiovascular status impact midazolam pharmacokinetics in critically ill children: An observational, prospective, controlled study. Pharmacol Res Perspect. 2022;10:e01004. 10.1002/prp2.1004 PMC9422629 36036654

[45] Uhing MR, Beno DWA, Jiyamapa-Serna VA, Chen Y, Galinsky RE, Hall SD, et al. The effect of anesthesia and surgery on CYP3A activity in rats. Drug Metab Dispos. 2004;32:1325–30. 10.1124/dmd.104.000927 15319324

[46] Watson J, Ninh MK, Ashford S, Cornett EM, Kaye AD, Urits I, et al. Anesthesia medications and interaction with chemotherapeutic agents. Oncol Ther. 2021;9:121–38. 10.1007/s40487-021-00149-1 PMC8140172 33861416

[47] Gelotte CK, Parasrampuria DA, Zimmerman BA. Single-dose pharmacokinetics and metabolism of the oral decongestant phenylephrine HCl in children and adolescents. Pulm Ther. 2023;9:139–50. 10.1007/s41030-022-00206-8 PMC9931972 36480111

[48] Sivak EL, Davis PJ. Review of the efficacy and safety of remifentanil for the prevention and treatment of pain during and after procedures and surgery. Local Reg Anesth. 2010;3:35–43. 10.2147/lra.s7709 PMC3417946 22915867

[49] Avelino SOM, Neves RM, Sobral-Silva LA, Tango RN, Federico CA, Vegian MRC, et al. Evaluation of the effects of radiation therapy on muscle contractibility and skin healing: An experimental study of the cancer treatment implications. Life (Basel). 2023;13:1838. 10.3390/life13091838 PMC10532574 37763242

[50] Kumari S, Mukherjee S, Sinha D, Abdisalaam S, Krishnan S, Asaithamby A. Immunomodulatory Effects of Radiotherapy. Int J Mol Sci. 2020;21(21):8151. 10.3390/ijms21218151 PMC7663574 33142765

[51] Young Park W, Thompson JS, Lee KK. Effect of epidural anesthesia and analgesia on perioperative outcome. Ann Surg. 2001;234:560–71. 10.1097/00000658-200110000-00015 PMC1422079 11573049

